# The low abundance of CpG in the SARS-CoV-2 genome is not an evolutionarily signature of ZAP

**DOI:** 10.1038/s41598-022-06046-5

**Published:** 2022-02-14

**Authors:** Ali Afrasiabi, Hamid Alinejad-Rokny, Azad Khosh, Mostafa Rahnama, Nigel Lovell, Zhenming Xu, Diako Ebrahimi

**Affiliations:** 1 BioMedical Machine Learning Lab, The Graduate School of Biomedical Engineering, UNSW Sydney, Sydney, NSW 2052 Australia; 2 UNSW Data Science Hub, The University of New South Wales, Sydney, NSW 2052 Australia; 3 Health Data Analytics Program, AI-Enabled Processes (AIP) Research Centre, Macquarie University, Sydney, 2109 Australia; 4 Department of Biology, Yazd University, Yazd, 8915818411 Iran; 5 Department of Plant Pathology, University of Kentucky, Lexington, KY 40546 USA; 6 The Graduate School of Biomedical Engineering, UNSW Sydney, Sydney, NSW 2052 Australia; 7 Department of Microbiology, Immunology and Molecular Genetics, University of Texas Health Science Center at San Antonio, San Antonio, TX 78229 USA; 8 Texas Biomedical Research Institute, San Antonio, TX 78227 USA

**Keywords:** Computational biology and bioinformatics, Genetics

## Abstract

The zinc finger antiviral protein (ZAP) is known to restrict viral replication by binding to the CpG rich regions of viral RNA, and subsequently inducing viral RNA degradation. This enzyme has recently been shown to be capable of restricting SARS-CoV-2. These data have led to the hypothesis that the low abundance of CpG in the SARS-CoV-2 genome is due to an evolutionary pressure exerted by the host ZAP. To investigate this hypothesis, we performed a detailed analysis of many coronavirus sequences and ZAP RNA binding preference data. Our analyses showed neither evidence for an evolutionary pressure acting specifically on CpG dinucleotides, nor a link between the activity of ZAP and the low CpG abundance of the SARS-CoV-2 genome.

## Introduction

Severe Acute Respiratory Syndrome Coronavirus 2 (SARS-CoV-2), the causative agent of coronavirus disease 2019 (COVID-19) pandemic, has a ~ 30 kb single-stranded positive RNA (+ssRNA) genome, which is one of the largest known viral RNA genomes^1^. The SARS-CoV-2 RNA has unique genomic features with likely roles in the high pathogenicity and cross-species transmission of this virus^2,3^. An expansive view of the SARS-CoV-2 genomic features is an essential step to improve our current understanding of the evolutionary path of this virus. One of these unique features is the low abundance of CpG in the SARS-CoV-2 genome^4–8^. CpG depletion is a well-known phenomenon in viruses particularly those with RNA genomes^9,10^. It has been reported that the CpG composition of + ssRNA viral genomes often mimics the CpG content of their hosts, however the underlying molecular mechanisms are not well understood^11^. One of the suggested mechanisms is DNA cytosine methylation-induced deamination^11–13^. However, SARS-CoV-2 does not have a DNA stage, thus this mechanism is not likely to be relevant. Another suggested mechanism is recognition of CpG sites within viral RNA by the host RNA-binding protein ZAP (CCCH-type zinc finger antiviral protein)^8^. ZAP is known to restrict the replication of a broad range of viruses including SARS-CoV-2 by binding to the CpG rich regions of viral RNA, and subsequently inducing viral RNA degradation^14–17^. The low CpG content of RNA viruses have been proposed to be linked to this mechanism^14–16^.

Xia^8^ and Wei et al.^18^ have proposed that the low CpG content of SARS-CoV-2 might be due to an evolutionary pressure from ZAP^8^. Among the coronaviruses studied in Xia’s study, those isolated from canines were shown to have the most CpG depleted genomes. Therefore, it was postulated that dogs may have been the intermediate species for the emergence of SARS-CoV-2. This hypothesis is based on two assumptions, which are not likely to be correct: first, only the frequency of CpG (but not those of other 15 dinucleotides ApA, ApC, …, UpU) is sufficient to make inferences about the origin of viruses, and second, ZAP is the main source of low CpG abundance in the SARS-CoV-2 genome. A number of follow up studies challenged Xia’s methodology and conclusion. For instance, Pollock et al. repeated Xia’s analysis using a larger number of SARS-CoV-2 related viruses and found that CpG deficiency is not specific to dog coronaviruses or SASR-CoV-2, and it is observed in pangolin coronaviruses and to a greater extent in pangolin pestiviruses^5^. Moreover, modeling of the binding affinity of ACE2 and SARS-CoV-2 Spike protein in 410 vertebrates showed a low score of susceptibility to SARS-CoV-2 infection for dogs^19^. This finding was also confirmed by viral replication experiments^20^. Digard et al. showed that CpG abundance varies significantly across the SARS-CoV-2 genome, with envelope and ORF10 not showing CpG depletion^21^. They showed that the CpG levels of SARS-CoV and SARS-CoV-2 envelope sequences are even higher than those of envelope from other human coronaviruses. Using a phylogenetic analysis, the authors argued that these genomic composition changes are more likely to be an ancestrally-driven traits related to the origin of these viruses in bats, not due to a post-zoonotic transfer selection force. These data suggest that the overall CpG content alone is not a reliable index for inferring the host origin of viruses^21^. Furthermore, Xia et al.^8^ and Wei et al*.*^18^ based their argument on the baseline levels of ZAP in the SARS-CoV-2 intermediate host tissues. It is reasonable to argue that ZAP expressions in healthy/uninfected cells/tissues do not reflect the ZAP expression levels during viral infection^22–24^.

Here, we perform a detailed analysis of multiple data sets including the representations of short sequence motifs in viral genomes and patterns of ZAP binding to viral RNA to investigate the role of ZAP in reducing the SARS-CoV-2 CpG level. Our analyses show that the representations of almost all dinucleotides, not only CpG, are different in the SARS-CoV-2 genome compared to the genomes of other coronaviruses. For example, UpC, and ApU are all represented at significantly lower levels in the SARS-CoV-2 genome compared to the SARS-CoV genomes. Our analyses indicate that not only the CpG motifs preferentially targeted by ZAP but also those not often recognized by ZAP have lower representation in SARS-CoV-2 compared to SARS-CoV. Altogether, our results provide multiple lines of evidence against the role of ZAP in the evolution of the SARS-CoV-2 genome.

## Methods

### Viral sequences

**Figure 1:**
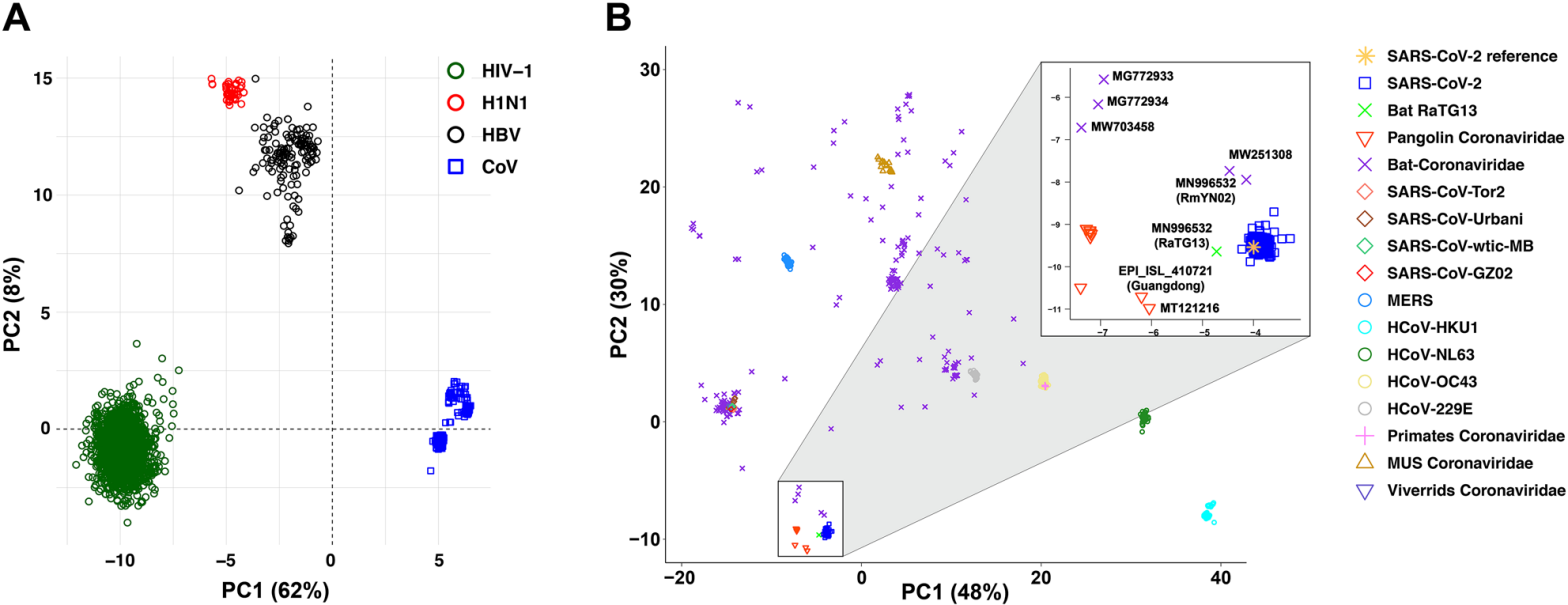
PCA of viral motifs representations. D-values of all dinucleotide, trinucleotide and tetranucleotide motifs in all viral sequences form a matrix, which is used as an input for PCA. (**A**) PC1-PC2 plot shows four clusters, one for each virus family: H1N1, coronaviruses (CoV), HBV, and HIV-1. (**B**) PC1-PC2 plot classifies coronaviruses into two clusters. All 664 SARS-CoV-2 (including reference sequence), Bat-RaTG13, RmYN02, 4 Bat-Coronaviridae viruses (MW703458, MW251308, MG772933, MG772934), and 10 Pangolin-Coronaviridae (EPI_ISL_410721, EPI_ISL_410539, EPI_ISL_410542, EPI_ISL_410543, EPI_ISL_410538, EPI_ISL_410541, EPI_ISL_410540, MT040336.1, MT040333.1, MT121216.1) formed a cluster (SARS-CoV-2-like group), which is separated from the rest of coronavirus sequences including Human coronavirus 229E, Bat-Coronaviridae, Human coronavirus HKU1, Murine coronavirus, MERS coronavirus, Human coronavirus NL63, Human coronavirus OC43, Primates-Coronaviridae, SARS coronavirus Tor2, SARS coronavirus Ubani, Viverrids-Coronaviridae, SARS coronavirus wtic_MB, SARS coronavirus GZ02. SARS-CoV-2-like are highlighted with a square.

For the analysis presented in Fig. 1A, we used full-length sequences of 3967 coronaviruses, 2021 HIV-1, 91 Flu H1N1, and 141 HBV, totaling 6220 sequences from GenBank. For the analysis presented in Fig. 1B, we obtained a total of 1546 full-length coronavirus genomic sequences (323 human SARS-CoV, 10 viverridis coronaviruses, 190 bat coronaviruses, 41 mus SARS-CoV, 5 primates coronaviruses, 256 MERS coronaviruses, 88 murine coronaviruses, 10 pangolin coronaviruses, and 664 SARS-CoV-2). To ensure that our analysis is not affected by variations accumulated in the SARS-CoV-2 genome over time, we only used 664 SARS-CoV-2 sequences reported before January 31st, 2020 (Supplementary Table 1).

### Analysis of motif representation

We quantified the representation of short sequence motifs (di-, tri- and tetra-nucleotides) in all viral genomes using our previously reported Markov-based representation (D-value) method^25–27^. Briefly, motif representation (D-value) is defined as the ratio of the observed frequency (*P*_obs_) of a motif over its expected frequency (*P*_exp_). *P*_obs_ is simply the observed relative frequency of the motif. *P*_exp_ is quantified using the frequency of the motif in the sequence and the frequencies of the smaller constituting motifs^12^. An example of a D-value for the tri-nucleotide motif ACG is given in Eq. (1).

1 $$D = \frac{{ACG_{obs} }}{{ACG_{exp} }} = \frac{{ACG_{obs} \times C_{obs} }}{{AC_{obs} \times CG_{obs} }}$$

For each analysis, we arranged the D-values of all dinucleotide, trinucleotide, and tetranucleotide motifs of all sequences in a data matrix. The matrices were then analyzed by principal component analysis (PCA). We performed two separate PCAs. The first analysis was done on 3967 coronaviruses 2021 HIV-1, 91 Flu H1N1, and 141 HBV sequences to demonstrate that motif representation data can be used to separate different virus families. The second PCA was performed on 1546 coronavirus sequences to identify viruses whose genome show high similarities to the SARS-CoV-2 genome. To quantify similarities (i.e. distances from the SARS-CoV-2 cluster) in the principal component space, we used Mahalanobis distance^28^. Further, Mann–Whitney test was used to determine the difference in the median of sequence motif representation (D-value) between the SARS-CoV-2-like group (SARS-CoV-2 and closely related coronaviruses) and viruses of the SARS-CoV group.

### Analysis of association between CpG abundance and ZAP

To investigate the association of ZAP binding regions in the viral genomes with the number of CpGs co-located with these binding regions, we used the publicly available datasets of cross-linking immunoprecipitation (CLIP-seq) reported for ZAP. We obtained the processed ZAP CLIP-seq data (density of reads aligned to the genome) for wild type JEV (Japanese Encephalitis Virus) and wild type HIV^16,29^. We then calculated the CpG density across the JEV and HIV genomes using a sliding window analysis method. We used 200 bp window and 1Bp sliding for JEV and 250 bp window and 1Bp sliding for HIV.

The motif C(n7)G(n)CG has been demonstrated to be the optimal binding motif for Mouse ZAP^30^. To further investigate the role of ZAP in reducing CpG abundance in shaping the genome of SARS-CoV-2, we analyzed the abundance of C(n7)G(n)CG in SARS-CoV-2-like viruses. We used the abundance of motif C(n7)C(n)CG as a negative control in our analysis. Mann–Whitney test was used to determine the significance of difference in the median of these two motifs in the SARS-CoV-2 genome.

## Results

### Analysis of motif representations using PCA

We applied principal component analysis (PCA) on motif representation (D-value) matrices to interrogate similarities between and within virus families (Supplementary Table 2 and 3). Figure 1A shows the PCA of all four virus groups studied here, and Fig. 1B shows the results of PCA on coronaviruses only (see “Methods” section for details). As indicated in Fig. 1A, all of the four virus families HIN1, HBV, HIV-1 and coronaviruses (CoV) are separated using the first two PCs. PCA of only coronavirus sequences is depicted in Fig. 1B. All groups of coronaviruses are clearly separated from each other except Bat-Coronaviridae viruses which are diverse and form multiple clusters. All 664 SARS-CoV-2 (including reference sequence) and several bat and pangolin coronaviruses formed a cluster (SARS-CoV-2-like group shown in a square in Fig. 1B; see Supplementary Table 4), which was separated from the rest of coronavirus sequences including human coronaviruses 229E, the rest of bat coronaviruses, human coronaviruses HKU1, MERS, murine coronaviruses, human coronaviruses NL63, human coronaviruses OC43, primate coronaviruses, and SARS coronaviruses (Tor2, Urbani, Viverrids, Wtic-MB and GZ02, which we refer to as SARS-CoV here).

### Representation of dinucleotides in the genome of SARS-CoV-2-like and SARS-CoV viruses

**Figure 2:**
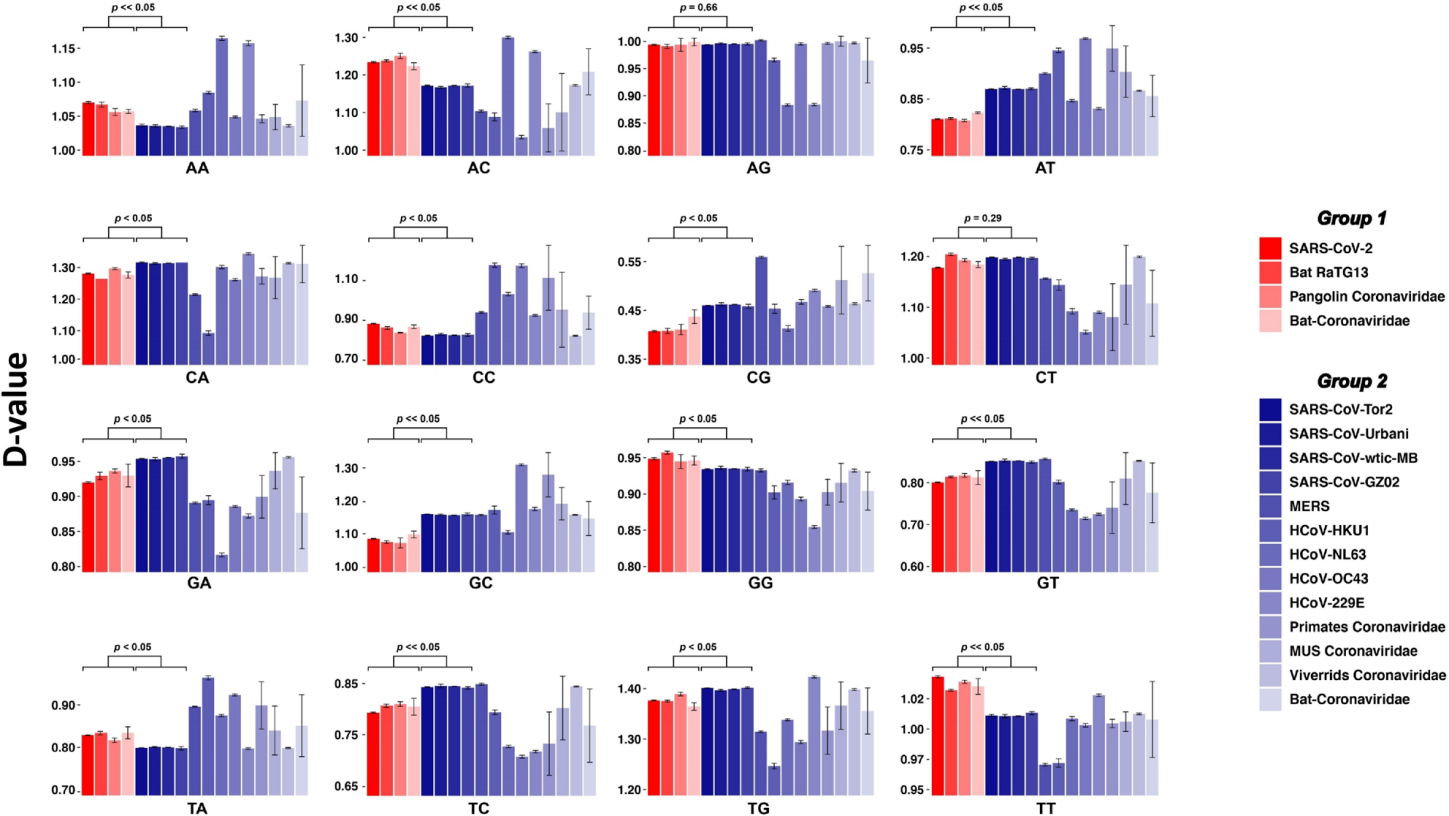
Comparison of dinucleotide motif representations between SRAS-CoV-2-like and SARS-CoV groups. D-values of each dinucleotide were compared between the two viral groups SARS-CoV-2-like and SARS-CoV. Mann–Whitney test was used to examine the difference in the median of D-values between the two coronavirus groups. D-value (motif representation) is defined as the ratio of the observed frequency (Pobs) of a motif over its expected frequency (Pexp). Pobs is simply the observed relative frequency of the motif. Pexp is quantified using the frequency of the motif in the sequence and the frequencies of the smaller constituting motifs.

As indicated in Fig. 1B, coronaviruses used in this study form a distinct group (SARS-CoV-2-like) based on their motif representations (D-values). We compared, for each dinucleotide, the median D-values of SARS-CoV-2-like (group 1) with SARS-CoV (group 2) (Fig. 2 and Supplementary Table 3). All dinucleotide motifs (not only CpG) except for ApG and CpU were significantly different between the two groups. There is an excess of ApA, ApC, CpC, GpG, UpA and UpU, and a deficit of ApU, CpA, GpA, GpC, GpU, UpC, UpG and CpG in group one (SARS-CoV-2-like) compared to group two (SARS-CoV).

### Analysis of ZAP binding to CpG sites

**Figure 3:**
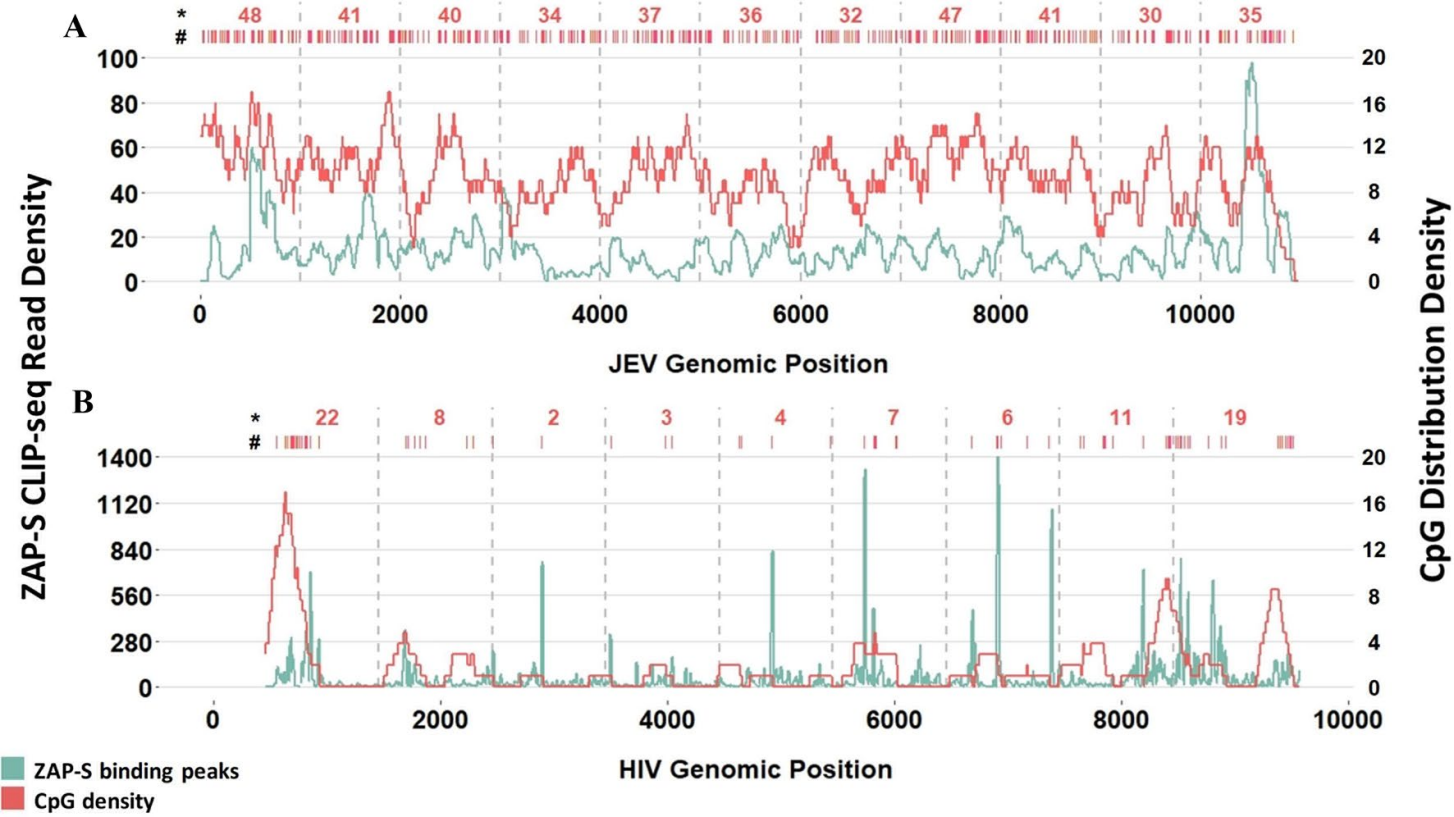
Co-location of ZAP binding regions and CpG motifs. Overlaying of the ZAP binding peaks and CpG densities in (**A**) JEV genome (Japanese Encephalitis Virus) and (**B**) HIV-1. The ZAP binding peaks (density of reads aligned to the genome) are estimated using a 250 bp sliding window moving by 1 bp along the viral genomes. The CpG density was calculated using the same sliding window analysis method, except we used a 200 bp window sliding by 1 bp in JEV and a 250 bp window sliding by 1pb in HIV-1. ZAP binding peaks and CpG densities are shown in green and red, respectively. # Location of CpGs. * Number of CpGs per 1 Kb.

To determine the CpG motif specificity of ZAP, we overlaid the ZAP CLIP-seq data and the CpG distribution of two viruses, HIV and JEV (Japanese Encephalitis Virus) (Fig. 3). We found no clear and consistent pattern of co-localization between ZAP binding regions and CpG sites in HIV and JEV genomes.

**Figure 4:**
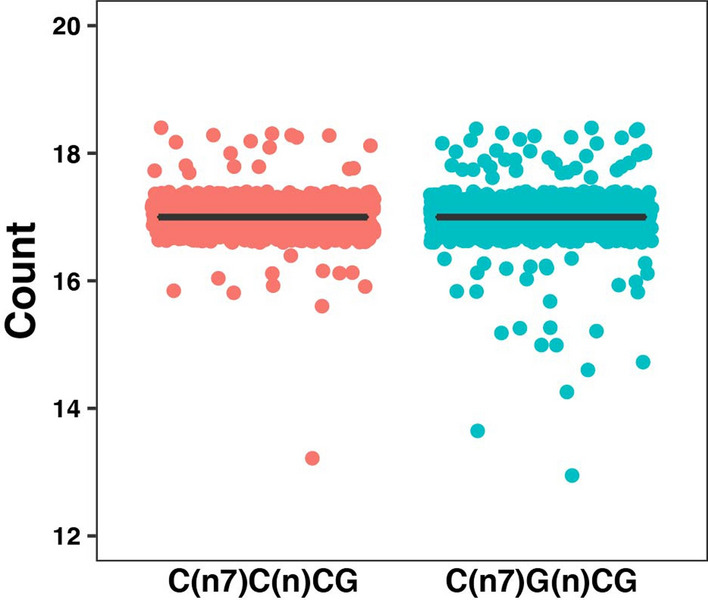
Comparison of the abundance of ZAP optimal binding motif C(n7)G(n)CG with the control motif C(n7)C(n)CG in viruses of SARS-CoV-2-like group. The abundance of ZAP optimal binding motif C(n7)G(n)CG was compared to C(n7)C(n)CG in the SARS-CoV-2-like group. The motif C(n7)C(n)CG was used here as a control. Mann–Whitney test was used to determine the difference in the median of abundance between these two motifs.

It has been shown that ZAP binds preferentially to C(n7)G(n)CG motifs^30^. To further investigate the role of ZAP binding in reducing the SARS-CoV-2 CpG level, we compared the relative frequency of C(n7)G(n)CG and C(n7)C(n)CG in SARS-CoV-2-like viruses (Supplementary Table 5). The ZAP preferred motif C(n7)G(n)CG and the motif C(n7)C(n)CG which is not a preferred ZAP binding motif have similar abundances (*p* value >  > 0.05, Fig. 4).

## Discussion

It is critical to understand the evolutionary trajectory of SARS-CoV-2 and determine the molecular mechanisms that have contributed to its pathogenicity and adaptation to human cells. This information can help prevent and/or better control future pandemics caused by coronaviruses. Previous studies have shown that CpG is depleted in the genome of SARS-CoV-2 and its related bat and pangolin coronaviruses. To better understand the source of CpG depletion in these viruses, the first step was to identify the sequences that are closely related to SARS-CoV-2. We have previously shown that an alignment-free method that uses the representation of short sequence motifs can precisely identify HIV-1 subtypes^31^. Here, we used the same method to investigate the similarities between and within virus families. This method successfully classified different virus families. As expected, it showed a separate cluster for SARS-CoV-2 with several bat and pangolin coronaviruses. Our analyses confirmed that SARS-CoV-2 and its closely related coronaviruses have a lower CpG content compared to other coronaviruses. However, we found that reduction in the representation of CpG was comparable to the reduction in GpC and ApT dinucleotides. More importantly, changes in motif representation are not exclusive to CpG. Most of the dinucleotides have significantly different representations in the viruses of the SARS-CoV-2-like group compared to the viruses in the SARS-CoV group. Altogether, our data suggest that the low abundance of CpG is not exclusive to the SARS-CoV-2 genome and is a general feature of several bat and pangolin coronaviruses. Most importantly, changes in the abundance of dinucleotides are not specific to CpG. Therefore, CpG reduction is likely part of a global genomic difference rather than being a signature of an exclusive selection force against CpG motifs. These data are in line with a study by Di Giallonardo et al. showing that dinucleotide composition of RNA viruses is shaped by the virus family not their hosts^32^.

Immune evasion is one of the mechanisms proposed to explain the low CpG abundance in viral genomes including coronavirus sequences^9,11,33–36^. Assuming that this hypothesis is true, one would expect to observe a significant CpG depletion in the genome of SARS-CoV-2 to justify its high transmission rate and pathogenicity. By contrast, there is a little difference between the SARS-CoV-2-like and SARS-CoV viruses in terms of CpG abundance. The average CpG counts per kilobase (Kb) is 14.7 and 19.3 in group one (SARS-CoV-2-like) and group two (SARS-CoV) viruses, respectively. This means, on average, compared to SARS-CoV, SARS-CoV-2 has ~ 4.6 less CpGs per 1 Kb. It is unlikely that such a marginal CpG difference plays a critical role in SARS-CoV-2 immune evasion. Importantly, a previous study showed no correlation between the pathogenicity and global CpG content of coronaviruses infecting humans^4^. Furthermore, it has been shown that the CpG content of SARS-CoV-2 sequences is highly variable across the viral genome. In some regions of the SARS-CoV-2 genome such as envelop, not only CpG is not depleted, but it is more abundant compared to some of the other coronaviruses. This suggests the global CpG content is unlikely to be a vital genomic feature of the SARS-CoV-2 genome with a role in immune evasion^21^. Altogether, the overall CpG reduction in the SARS-CoV-2 genome is likely unrelated to the pressure imposed on the virus by the innate immune system.

Among the components of the human innate immune system, ZAP has been shown to play a key role in the inhibition of RNA viruses by binding to CpG containing sequences and recruiting a RNA-degradation machinery^16^. Xia’s study postulates that the source of SARS-CoV-2 is a bat coronavirus whose genome underwent further CpG reduction by ZAP after the virus infected an intermediate species with a high ZAP expression level (possibly a canine tissue). It was suggested that ZAP-induced CpG depletion of viral RNA in this intermediate species led to the generation of SARS-CoV-2, which was able to infect human cells^8^. Our analyses show a poor association between the abundance of CpG across viral genomes and the location of ZAP binding peaks. These data suggest that the binding of ZAP to viral RNA is likely not governed by the global CpG abundance of viral genomes. In support of our results, a study has shown that ZAP inhibition is independent of the viral CpG content^37^. Additionally, a recent study shows that the location and sequence context of CpGs but not the overall CpG abundance of viral genome play a role in inducing ZAP antiviral activity^17^. Moreover, one of the mechanisms by which ZAP inhibits viruses is through the suppression of viral mRNA translation via blocking eIF4A^15^, which is independent of ZAP binding to viral RNA. Although it has been shown that ZAP is capable of inhibiting SARS-CoV-2 in vitro^38^, there is no evidence to support a role for ZAP in reducing the CpG level of the SARS-CoV-2 genome and shaping the genome of this virus.

A previous study has shown that mouse ZAP preferentially binds to C(n7)G(n)CG motifs where n: A, C, G, or U^30^. Assuming that human ZAP has the same motif preference and that it has induced an evolutionary pressure on the SARS-CoV-2 genome, one would expect the relative abundance of C(n7)G(n)CG to be lower than a non-ZAP binding motif (e.g. C(n7)C(n)CG) in the SARS-CoV-2 genome. Our analysis does not show a significant difference between the abundance of these motifs in the SARS-CoV-2 genome. This may provide yet another line of evidence against the role of ZAP in lowering the CpG content of SARS-CoV-2 genome.

We note that some of the studies of the evolutionary footprint of host immune mechanisms on viral genomes, focus merely on specific motifs, and ignore the overall composition of viral genomes. In many cases, this can lead to gross misinterpretation of data. For example, a phylogeny effect can be misinterpreted as an evolutionary signature. To better understand the role of ZAP and other restriction factors in the inhibition and/or evolution of viruses, a global analysis of viral genomic composition is needed. Differences observed in 14 out of 16 dinucleotides (i.e. not only CpG) point to general mechanism(s) with a global impact on the overall composition of SARS-CoV-2 genome. One of the mechanisms could be oxidative stress, although there is currently no data to support this hypothesis. Viral infection is often associated with oxidative stress, which results in producing reactive oxygen species (ROS)^39^. It has been shown that coronavirus infection causes a high level of ROS production in host cells^40^. Nucleotides, particularly guanine, are more prone to the oxidative damage caused by ROS, which oxidize guanine to 8-oxyguanine in both DNA and RNA^41^. It is known that 8-oxyguanine has a similar affinity for binding to adenine and cytosine^42^. There is a possibility that during the SARS-CoV-2 replication process^1^, which includes synthesizing a negative strand from the genome followed by making a positive strand using the newly synthesized negative strand, G is substituted with U. The lower representations of UpG and GpA accompanied with higher representations of UpU and UpA in the viruses of SARS-CoV-2-like group might be due to G > U mutations induced by oxidative stress. Nevertheless, there is currently no data to support this hypothesis.

In summary, we performed several independent analyses to determine if ZAP played a role in the emergence of SARS-CoV-2. Our analyses found no evidence to suggest that ZAP exerts an evolutionary pressure on the SARS-CoV-2 genome by targeting its CpG motifs.

## Supplementary Information

Supplementary Information.

## Data Availability

The data underlying this article are available at the online supplementary material.
